# Correlated Spatio-Temporal Data Collection in Wireless Sensor Networks Based on Low Rank Matrix Approximation and Optimized Node Sampling

**DOI:** 10.3390/s141223137

**Published:** 2014-12-05

**Authors:** Xinglin Piao, Yongli Hu, Yanfeng Sun, Baocai Yin, Junbin Gao

**Affiliations:** 1 Beijing Key Laboratory of Multimedia and Intelligent Software Technology, College of Metropolitan Transportation, Beijing University of Technology, Pingleyuan 100, Chaoyang District, Beijing 100124, China; E-Mails: piaoxinglin1987@gmail.com (X.P.); huyongli@bjut.edu.cn (Y.H.); ybc@bjut.edu.cn (B.Y.); 2 School of Computing and Mathematics, Charles Sturt University, Bathurst, NSW 2795, Australia; E-Mail: jbgao@csu.edu.au

**Keywords:** wireless sensor networks, data collection, low rank matrix approximation

## Abstract

The emerging low rank matrix approximation (LRMA) method provides an energy efficient scheme for data collection in wireless sensor networks (WSNs) by randomly sampling a subset of sensor nodes for data sensing. However, the existing LRMA based methods generally underutilize the spatial or temporal correlation of the sensing data, resulting in uneven energy consumption and thus shortening the network lifetime. In this paper, we propose a correlated spatio-temporal data collection method for WSNs based on LRMA. In the proposed method, both the temporal consistence and the spatial correlation of the sensing data are simultaneously integrated under a new LRMA model. Moreover, the network energy consumption issue is considered in the node sampling procedure. We use Gini index to measure both the spatial distribution of the selected nodes and the evenness of the network energy status, then formulate and resolve an optimization problem to achieve optimized node sampling. The proposed method is evaluated on both the simulated and real wireless networks and compared with state-of-the-art methods. The experimental results show the proposed method efficiently reduces the energy consumption of network and prolongs the network lifetime with high data recovery accuracy and good stability.

## 1. Introduction

Data collection is a fundamental application of wireless sensor networks (WSNs). For example, in the environmental monitoring scenario, the physical quantities, such as temperature, humidity and light illumination, *etc.* are usually sensed and transmitted from sensor nodes to sink nodes through multi-hop routing [1]. Since the sensor nodes usually have limited computing ability and power supply, a primary goal of data collection is to obtain the sensing data at required accuracy with least energy consumption and thus to prolong the network lifetime.

**Figure 1.**
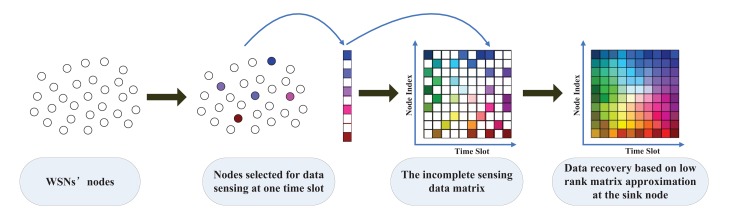
The correlated spatio-temporal data collection method based on LRMA and optimized node sampling.

Since the physical quantities describing natural phenomena are generally locally consistent and WSNs nodes are often deployed redundantly to get complete environmental monitoring data, the sensing data often have high spatial or temporal correlation [2]. Thus many researchers utilize the spatial and temporal correlation to reduce the network energy consumption by delivering part of sensing data or their aggregation, such as distributed source coding techniques [3–5], in-network collaborative wavelet transform methods [6,7] and clustered data aggregation methods [8–11]. Although these methods can save certain data communication cost, they would bring extra computational overheads for sensor nodes and so demand considerable sensing cost. Recently the low rank matrix approximation (LRMA) method [12,13] provides a new scheme for data collection in WSNs [14–16]. The main idea of the LRMA based method is that the sensing data of WSNs are regarded as a low rank matrix and recovered from a subset sensing data. As demonstrated in Figure 1, the data collection consists of two stages: the data sensing stage and the data recovering stage. At the data sensing stage, instead of using all sensor nodes, a subset of sensor nodes which are the shaded ones in the second subfigure is randomly selected to sense physical quantity and deliver the sensing data to the sink node at each data collection round. At the data recovering stage, the sink node receives these incomplete sensing data over some data collection rounds shown in the third subfigure in which the shaded entries represent the valid sensing data and the white entries are unknown. And then we could use them to recover the complete data by LRMA method. As the LRMA based data collection methods reduce either the data communication cost or the data sensing cost, high energy efficiency can be achieved even with an increased computation cost at the sink node.

However, the current LRMA based data collection methods [14–16] also suffer from certain inefficiency both at the data sensing stage and the data recovering stage. At the data sensing stage, a random sampling scheme for the selection of sensing nodes is adopted in the current methods [14–16]. According to the LRMA theory [13], the uniformly random sampling method is an optimized sampling way to get good matrix complement result. However, the ideal random sampling method will result in uneven energy distribution of the network as the many-to-one data collection pattern, and thus shorten the network lifetime. At the data recovering stage, most of the current methods do not explore and utilize the spatial or temporal correlation of the sensing data. This could be the contributing factor to the certain setback of these methods, such as low data recovery accuracy, and lacking robustness to noises and outliers. Therefore, to improve the efficiency of the current LRMA based data collection methods, we always face two challenges. On the one hand, we should find out a proper way to formulate the relation between two important components in the data sensing procedure, the node sampling scheme and the scheme of estimating energy status of the network. Generally, the former has direct effect on the data recovery accuracy, and the later can determine the lifetime of the network. On the other hand, the main obstacle is how to represent the spatial and temporal relations of the incomplete node sensing data and further integrate these constraints to the current LRMA data recovery model. In this paper, we present a new data collection method based on LRMA, which improves the performance of the current method both at the data sensing and data recovering stages. At the data sensing stage, we propose to use Gini index to measure the spatial distributions of the selected sensing nodes and the evenness of energy status of the network. Under this measurement, the selection of sensing nodes is formulated as an optimization problem with the constraint of the network energy status. At the recovering stage, observing that the sensing data from a sensor generally has temporal consistency and the sensing data from different nodes has spatial correlation excepting for some noise and outliers. We give the representations of the spatial and temporal relationship of the node sensing data and add these constraints into the basic LRMA model and construct a correlated spatio-temporal constrained LRMA model (ST-LRMA). Then the ST-LRMA model is solved by an iteration method.

The proposed ST-LRMA based data collection method is tested on both simulated and real wireless networks compared with other relevant methods. The experimental results demonstrate the proposed method achieves better data recovery accuracy and improves energy efficiency to prolong network lifetime. Additionally, it is robust to noise and outliers.

The main contributions of this paper are:

(1) We propose a correlated spatio-temporal constrained low rank matrix approximation model, a novel data collection scheme in WSNs;
(2) We propose an optimized node sampling model for reducing energy consumption of WSNs, in which Gini Index is adopt and used as a tool to improve the current uniformly random sampling method.

The rest of the paper is organized as follows. In Section 2, we summarize the related work. Section 3 introduces the basic LRMA model based on random sampling. Section 4 presents the proposed ST-LRMA model. Section 5 elaborates the new optimized node sampling method. We will show the experimental results of our proposed methods compared with state-of-the-art methods, such as basic LRMA model [14], T-LRMA model [15,16] and S-LRMA model in Section 6. Section 7 concludes the paper with a discussion on future research.

## 2. Related Works

In many data collection scenarios in WSNs, such as environmental monitoring [17], to fully sense physical quantities efficiently, it is necessary to transmit all sensing data to the sink node correctly and accurately. Doing so would consume too much energy in the networks. However, it is unnecessary to get the entire sensing data, given that there exists high correlation in the sensing data. Hence, the best strategy is to explore energy efficient data collection methods which approximately get the sensing data with a given accuracy.

The conventional approximate data collection methods include data aggregation techniques, distributed source coding and collaborative in-network compression methods. Data aggregation techniques [8–11] formulate the network sensor nodes into a hierarchical structure like trees or clusters. Generally the sensing nodes send the sensing data to the middle aggregator nodes. These aggregators collect data from multiple sensor nodes and aggregate the sensing data by aggregation functions, and then send the aggregated results to upper aggregators or sink nodes. Only a subset of nodes is involved in the communication with an aggregator and thus the overall energy consumption of the network during data collection is reduced. Distributed source coding schemes are based on the Slepian-Wolf coding theory [3–5]. In these methods, the sensor nodes are modeled as correlated sources of information and the dependency between the measured data are exploited to obtain data compression. Collaborative in-network compression methods can be viewed as an extension of traditional transforms in signal processing to sensor networks with irregular topology. Nodes can communicate with their neighbors and spatial correlation is exploited by collaborative transforms such as the distributed wavelet transform [6] and the graph wavelet transform [7].

Although the above mentioned conventional data collection methods are considered to be energy efficient and widely used in WSNs applications, they have their respective advantages in different aspects and the energy efficiency of these methods is relative to specific application scenarios. For example, the aggregation methods generally depend on a particular routing protocol, and the source coding methods typically require exact knowledge of the correlation between the measurements of different sensor nodes. It is highly desired to investigate the generic data collection method with high energy efficiency. The typical representatives are the recently proposed compressive sensing (CS) based methods [18–22] and the LRMA based methods [14–16].

The CS based methods provide two features of universal sampling and decentralized encoding, which make it a new paradigm for data collection in WSNs. In a single-hop network, a universal compressive wireless sensing method is proposed to deliver the sensing data by synchronized amplitude-modulated analog transmissions to the fusion center [23]. The sparsity of the sensing data and the CS decoding algorithms are discussed for data collection application in [24]. The first complete scheme to apply compressive sensing theory to data collection in large scale sensor networks is presented in [18]. Then an adaptive compressive sensing based data collection method is proposed in [19]. By maximizing information gain per energy cost during each measurement, the method adaptively collects data in an energy efficient way. However, during each measurement, the projected vector is obtained by solving an NP-hard optimization problem, which brings in relatively high computational and communication overhead. Energy efficiency of applying CS to data collection was investigated in [20], aiming at minimizing energy consumption through combining compressed aggregation with routing protocol. The optimization problem is proved to be of NP-completeness and both optimized and nearly-optimized solutions are given. It is proved that CS based data collection methods reduce the communication cost with high efficiency, but the data decoding usually involves high computation and the energy efficiency generally predominates in large scale WSNs. In addition, during the process of data collection, it generally demands complete sampling for all nodes in each round. Once there exist data missing, error or outliers, the result of data reconstruction would be greatly compromised.

As an efficient data recovery method, the LRMA model is widely used in many scenarios, and especially the fast algorithm for the solution of low rank minimal optimization was proposed in [25]. A CS method combined with low rank approximation was proposed to interpolate the Internet traffic matrices, in which the spatio-temporal properties of the matrices were considered [26]. The low rank completion method was adopted to reconstruct the fingerprint from a small subset of training samples [27]. The spatial correlation structure was combined with low rank completion for Received Signal Strength Indication (RSSI) recovery [28]. The basic LRMA model is first introduced to reduce energy consumption in data collection in [14]. Compared with the globally random sampling of the CS based data collection method which realizes energy efficiency by reducing the communication cost, the random sampling scheme of the LRMA based method reduces both the communication and sensing cost at nodes, which is the main virtue of the LRMA based method. However, in the basic LRMA model, the spatial or temporal correlation of the sensing data is not exploited. To obtain better data recovery results, a temporally constrained LRMA model for data collection was proposed in [15]. The similar idea was shared by our previous work [16]. However, the spatial properties of the sensing data are not well explored in any currently existing methods. In this paper, we consider both the temporal and spatial correlation of the sensing data simultaneously to propose an improved LRMA based data collection method. Furthermore, the issues of optimizing sensing node selection and balancing network energy distribution are also discussed.

## 3. The Basic LRMA Based Data Collection Method

In this section, we formulate the basic LRMA model for data collection in WSNs from the low rank theory [13]. For a wireless network with *N* sensor nodes, the *T* rounds data collection will form a matrix of measurements **X** ∈ *R^N^*^×^*^T^*. As shown in Figure 1, at each data collection round, the LRMA based method randomly selects a subset of sensor nodes for data sensing. Thus the measurement matrix is generally incomplete, *i.e.*, only the positions corresponding to the selected nodes offer valid sensing data. Here we adopt a mask operator 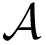 (·) [13] to represent the subset sampling procedure:

(1) A(X)=M.

where **M** is an incomplete matrix with only a sparse set of elements with valid sensing values at relevant positions. For the sake of clarity, the operator 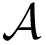
(·) can be specified as an element-wise matrix product as follows [13]:

(2) A(X)=Q⊙X.

where ⊙ denotes the Hadamard product of two matrices *i.e.*, **M**(*i, j*) = **Q**(*i, j*)**X**(*i, j*). **Q** is a *N* × *T* matrix defined as by the following form [14]:

(3) $$\text{Q}(i,j)=\{\begin{array}{ll} 1, & \text{if the}\;i\text{th node has valid sensing data at the}\;j\text{th time slot}.\\ 0, & \text{otherwise}. \end{array}$$

Generally, the measurements from one node are continuous in time domain, which shows temporal correlation. On the other hand, the sensing data from adjacent nodes have similar values or the sensing data of a node can be represented by the measurements of its neighboring nodes, which shows spatial correlation. These spatio-temporal correlations bring the low rank property of the measurement matrix **X**. According to the low rank matrix completion theory [12,13], it is highly possible to recover a low-rank matrix from a subset of its entries, generally a random subset with enough elements. Thus, we can recover **X** by solving the following optimization problem:

(4) $$\begin{array}{lll} \text{X}* & =\begin{array}{c} \text{argmin}\\ \text{X} \end{array} & \text{rank}(\text{X})\\ & \text{subject to} & A(X)=\text{M}. \end{array}$$

This is the basic LRMA (B-LRMA) model [12,13] for data collection. In the representation of B-LRMA model, there is no constraint on the rows or columns continuity of **X**. That is, the intrinsic properties of the measurements, the spatial and temporal correlations, are not represented in this model. So to get better recovery result of **X**, we propose a correlated spatio-temporal LRMA model. It will be described in detail in the next section.

## 4. The ST-LRMA Based Data Collection Method

The sequential measurements from a sensor, a row of the measurement matrix, are correlative in time domain and generally behave consistently and smoothly except for some noise and outliers, so it is natural to require the recovered matrix to preserve this property. Based on this observation, we share the similar idea of [15,16] and introduce a temporal constraint into the B-LRMA model to build a temporal constrained LRMA model (T-LRMA) for maintaining the consistency and smoothness in row direction, the adjacent columns of the recovered measurements. We formulate the T-LRMA model as follows [15,16]:

(5) $$\begin{array}{lll} \text{X}* & =\begin{array}{c} \text{argmin}\\ \text{X} \end{array} & \text{rank}(\text{X})+\lambda_{1}{\left\|\text{XD}\right\|}_{F}^{2},\\ & \text{subject to} & A(\text{X})=\text{M}. \end{array}$$

where λ_1_ is a tunable parameter, and **D** is a matrix with the following form [15,16]:

(6) $$\text{D}={\left(\begin{array}{ccccc} -1 & 0 & 0 & \ldots & 0\\ 1 & -1 & 0 & \ldots & 0\\ 0 & 1 & -1 & \ldots & 0\\ \vdots & \vdots & \ddots & \ddots & \vdots \\ 0 & 0 & 0 & \ddots & -1\\ 0 & 0 & 0 & \ldots & 1 \end{array}\right)}_{T\times (T-1).}$$

${\left\|\text{XD}\right\|}_{F}^{2}$ represents Frobenius norm of difference matrix of **X** in row direction, and thus enforces the consistency of the columns of the recovered **X**.

In practice, the sequential consistency is a ubiquitous property for many physical data and its sparse representation is well studied by many researchers. A fused LASSO method was proposed for problems with features that can be ordered in sequence [29]. In this method, the sparsity of regression coefficients and the smoothness constraint of the successive coefficients are considered simultaneously. A spatial subspace clustering method (SpatSC), was proposed for classifying the drill pope spectral data [30]. In this method, each individual regression coefficient vector of the prototypic data is demanded to be sparse and considered to be similar to neighboring. An ordered subspace clustering (OSC) method was proposed to segment data drawn from a sequentially ordered union of subspaces was proposed in [31]. In this method, each sequential nature of sequential data is incorporated by its neighbor penalty term to enforce similarity. The idea of preserving sequential consistency is shared by these methods and our method, but we apply the sequential constraint on the recovered data instead of the sparse coefficients.

Except for the temporal correlation of measurements from one node, there also exists spatial correlation among the measurements from different sensor nodes. The fundamental argument for this point is that the physical quantities from natural environment generally have smooth distribution in spatial domain, for example, the temperature distribution in a monitoring area. Another reason for favoring the spatial correlation is that the sensor nodes are generally deployed redundantly, sometimes densely, in order to obtain complete sensing data, which brings the spatial correlation among the measurements, especially for adjacent nodes. The closer nodes usually have similar measurements. To describe this property of the measurements, we incorporate the spatial constraints into the B-LRMA model and propose a spatial constrained LRMA model (S-LRMA) as follows:

(7) $$\begin{array}{lll} \text{X}* & =\begin{array}{c} \text{argmin}\\ \text{X} \end{array} & \text{rank}(\text{X})+\lambda_{2}{\left\|\text{SX}\right\|}_{F}^{2},\\ & \text{subject to} & A(\text{X})=\text{M}. \end{array}$$

where λ_2_ is a tunable parameter, and **S** is a matrix representing the relation among the measurements from different nodes, the rows of the measurement matrix **X**.

It is critical to design a proper **S** to obtain good data recovery results. Generally, the spatial correlation of the sensing data depends on the spatial deployment of the nodes. Therefore, it is a straight forward way to use the spatial relation of the nodes to model the spatial correlation of the sensing data. In the ideal case, such as the simulated network in Section 6, the measurement of a node can be represented as the linear combination of its neighbors according to its adjacent relation. Thus, we can construct **S** from the combination coefficients directly. But in practice, the accurate positions of sensor nodes are usually not available. Additionally, in a complex sensing scenario, the variation of the physical quantities is not smoothly distributed in the spatial domain and the measurements are often interfered by the complicated environment. Hence, in most cases, a feasible way inferring the spatial correlation of the sensing data is to utilize the sensing data themselves.

Although the traditional clustering methods, such as KNN, can be used to obtain the relation of a set of data in Euclid Space, it is difficult to get good result for high-dimension sensing data. Given that manifold learning methods [32-34] have good performance in learning the hidden structure in high dimension data, we adopt the manifold clustering method, namely sparse manifold clustering (SMC) method [35], to get the spatial correlation of sensing data. Conveniently, we implement the SMC model for sensing data spatial clustering as follows.

Similar to the ideal case in the simulated network, the measurement of a node can be represented as the linear combination of the measurements of correlated nodes, *i.e.*, for a row **X***_i_*, *i* = 1, …, *N* of **X**, there exist *K* most correlated rows **X***_jk_*, *k* = 1, ‥, *K* s.t. ${\text{X}}_{i}={\text{∑}}_{k=1}^{K}w_{j_{k}^{i}}{\text{X}}_{j_{k}}$, where *j_k_* ≠ *i*. So the problem of constructing **S** is to find the suitable weights $w_{j_{k}^{i}}$, *k* = 1, …, *K* for each **X***_i_*, *i* = 1, …, *N*, which represent the intrinsic structure among the high dimension signals, the rows of the sensing data. According to SMC, for each **X***_i_*, *i* = 1, …, *N*, we firstly construct a new signal matrix **Y***^i^* from the measurement matrix **X** as the following form:

(8) $${\text{Y}}^{i}=[{\text{Y}}_{1}^{i},\ldots ,{\text{Y}}_{i-1}^{i},{\text{Y}}_{i+1}^{i},\ldots ,{\text{Y}}_{N}^{i}].$$

where ${\text{Y}}_{j}^{i}=\frac{{\text{X}}_{j}^{T}-{\text{X}}_{i}^{T}}{{\left\|{\text{X}}_{j}^{T}-{\text{X}}_{i}^{T}\right\|}_{2}}$, *j* = 1, …, *N* and *j* ≠ *i*. Then we construct a diagonal distance matrix **Z***^i^* with elements $\frac{{\left\|X_{j}^{T}-X_{i}^{T}\right\|}_{2}}{{\text{∑}}_{j\neq i}{\left\|X_{j}^{T}-X_{i}^{T}\right\|}_{2}}$, *j* = 1, …, *N* and *j* ≠ *i*. Therefore, we can get an optimized sparse coefficient **c***_i_* from the following optimization problem:

(9) $$\begin{array}{cccc} \min & \alpha {\left\|{\text{Z}}_{i}{\text{c}}_{i}\right\|}_{1}+\frac{1}{2}{\left\|{\text{Y}}_{i}{\text{c}}_{i}\right\|}_{2}^{2}, & \text{s.t}. & 1^{T}{\text{c}}_{i}=1. \end{array}$$

From the optimized **c***_i_*, we can get the spatial correlation weights $w_{j_{k}^{i}}$,*k*= 1, …, *K* by omitting some small elements less than the given threshold *ε*:

(10) $$w_{j_{k}^{i}}=\frac{c_{ij_{k}}/{\left\|{\text{X}}_{j_{k}}^{T}-{\text{X}}_{i}^{T}\right\|}_{2}}{\underset{\begin{array}{c} j\neq 1\\ |c_{ij}|>\varepsilon \end{array}}{\text{∑}}\left(c_{ij}/{\left\|{\text{X}}_{j}^{T}-{\text{X}}_{i}^{T}\right\|}_{2}\right)}.$$

Based on the spatial correlation weights $w_{j_{k}^{i}}$, *k* = 1, …, *K*, the spatial correlation matrix **S** can be formulated by the following element representation:

(11) $$\text{S}(i,j)=\{\begin{array}{ll} 1, & \text{if}i=j.\\ -w_{j_{k}^{i}}, & \text{if}j\neq i\text{and}j=j_{k}^{i},k=1,\ldots ,K.\\ 0, & \text{otherwise}. \end{array}$$

One should note that **S** constructed in such a way is based on the complete measurement matrix **X**. However, at the very beginning, we could only obtain the incomplete sensing data. Therefore, prior to recovering the sensing data, we could estimate the unknown measurements by some simple interpolation algorithms or the B-LRMA. From the estimated complete sensing data, we could construct the spatial correlation matrix **S**. According to our experimental results, the T-LRMA data recovery model in Equation (5) has better estimated accuracy than other algorithms. So, we use the optimized result of the T-LRMA model as the estimation of the unknown sensing data to construct **S**.

Having determined the temporal and spatial constraints **D** and **S**, we integrate the low rank approximation models in Equations (5) and (7) into a combined model to form a novel correlated sptaio-temporal constrained model, the ST-LRMA model:

(12) $$\begin{array}{lll} \text{X}* & =\begin{array}{c} \text{argmin}\\ \text{X} \end{array} & \text{rank}(\text{X})+\lambda_{1}{\left\|\text{XD}\right\|}_{F}^{2}+\lambda_{2}{\left\|\text{SX}\right\|}_{F}^{2},\\ & \text{subject to} & A(\text{X})=\text{M}. \end{array}$$

In general, a rank minimization problem like Equation (12) is NP-hard [12]. The common practice in solving Equation (12) is to replace the rank function with the so-called matrix nuclear norm ‖ · ‖_*_ which is defined as the sum of singular values of **X**. Thus Equation (12) can be converted into a convex optimization problem [12]. Leveraging the computation and memory resource in the sink node and the data recovering delay for large scale WSNs (**N** nodes), we adopt the approximate algorithm in [12], which is considered to have good efficiency with a relative small memory request. This method seeks for a solution with a fixed low rank *r*. The process can be described as follows. Suppose that the rank of targeted solution **X** is *r*. Then **X** admits a skinny SVD **X** = **UΣV***^T^*, where **U** is an *N* × *r* unitary matrix, **V** is a *T* × *r* unitary matrix and **Σ** is an *r*×*r* diagonal matrix containing all the singular values *σ_k_, k* = 1, 2,…, *r*, which are arranged in a decreasing order. We further write as **X** = **UΣV***^T^* = **LR***^T^*, where **L** = **UΣ**^1/2^ and **R** = **VΣ**^1/2^. So the model in Equation (12) can be replaced by the following constrained minimization model:

(13) $$\begin{array}{lll} \left(\text{L}*,\text{R}*\right) & =\begin{array}{c} \text{argmin}\\ \text{L},\text{R} \end{array} & \text{rank}(\text{L}{\text{R}}^{T})+\lambda_{1}{\left\|\text{L}{\text{R}}^{T}\text{D}\right\|}_{F}^{2}+\lambda_{2}{\left\|{\text{SLR}}^{T}\right\|}_{F}^{2},\\ & \text{subject to} & A(\text{L}{\text{R}}^{T})=\text{M}. \end{array}$$

The dimension of **L** and **R** are *N* × *r* and *T* × *r*, respectively. In practice, we can specify an estimate to the rank *r* of **X**, which could be larger than the actual rank of the targeted **X**. From the lemma in [12], if the restricted isometry property holds on 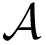
(**X**) and rank(**X**) < rank(**LR***^T^*), then Equation (13) is equivalent to the following model:

(14) $$\begin{array}{lll} \left(\text{L}*,\text{R}*\right) & =\begin{array}{c} \text{argmin}\\ \text{L},\text{R} \end{array} & {\left\|\text{L}\right\|}_{F}^{2}+{\left\|{\text{R}}^{T}\right\|}_{F}^{2}+\lambda_{1}{\left\|\text{L}{\text{R}}^{T}\text{D}\right\|}_{F}^{2}+\lambda_{2}{\left\|{\text{SLR}}^{T}\right\|}_{F}^{2},\\ & \text{subject to} & A(\text{L}{\text{R}}^{T})=\text{M}. \end{array}$$

By the method of Lagrange multipliers, the constrained optimization problem Equation (14) can be revised as the following unconstrained model:

(15) $$(\text{L}*,\text{R}*)=\begin{array}{c} \mathrm{argmin}\\ \text{L},\text{R} \end{array}{\left\|\text{L}\right\|}_{F}^{2}+{\left\|{\text{R}}^{T}\right\|}_{F}^{2}+\lambda_{1}{\left\|\text{L}{\text{R}}^{T}\text{D}\right\|}_{F}^{2}+\lambda_{2}{\left\|{\text{SLR}}^{T}\right\|}_{F}^{2}+\lambda_{3}{\left\|A(\text{L}{\text{R}}^{T})-\text{M}\right\|}_{F}^{2}.$$

where ${\left\|\text{A}({\text{LR}}^{T})-\text{M}\right\|}_{F}^{2}$represents the reconstruction error at the sampling subset **M** with an adjusting weight λ_3_. This is the final ST-LRMA model for data collection.

To solve problem Equation (15), we adopt an alternative iteration algorithm for **L** and **R**. Firstly, **L** is initialized randomly. Then optimize **R** by a linear least square method with fixing **L**. Note that the operator 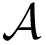
(·) confines the equation group onto the subset of the valid entries in **M**, so we use a subset of the equations in the linear least square aggression instead of the complete equation group. After **R** has been updated, we fix **R** and alternatively optimize **L** with respective to the new objective function. Repeat the above alternative iteration procedure until meeting the convergence condition or exceeding the maximal iteration number.

## 5. Optimized Node Sampling for Energy Efficient Data Collection

The LRMA based data collection depends on the random sampling measurements **M**. From the low rank completion theory [13], to get good recovery accuracy, the elements in **M** should better have a uniform distribution. Therefore, the selected nodes for sensing data should have uniform distribution in the deployed area, which is also the request for completely sensing the physical phenomena. However, as the many-to-one data collection pattern, this uniform node sampling method will bring unbalance energy distribution of the network and shorten its lifetime. To realize an energy efficient node selection at each data collection round, we propose an optimized node sampling method considering both the uniform random sampling and the network energy balance. In this method, we use Gini Index to measure the evenness of a random node sampling scheme and the network energy status and then find the optimized node sampling solution to meet the two-fold requests.

**Figure 2.**
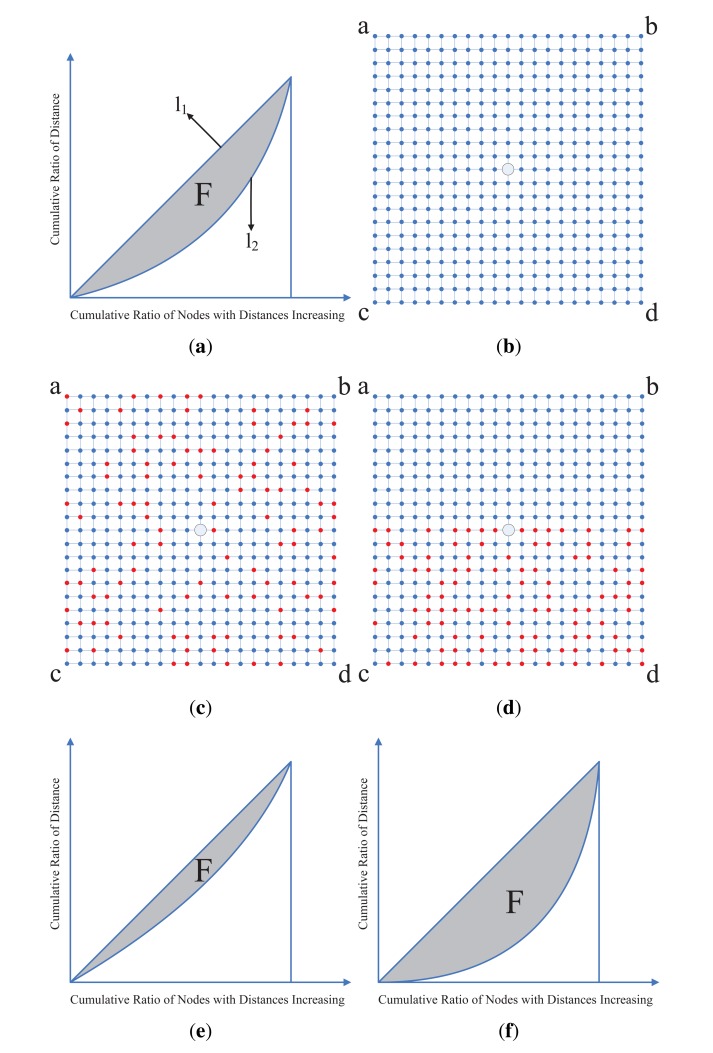
Two nodes sampling schemes in simulative networks and their Gini Index. (**a**) A demonstration of Gini Index; (**b**) A grid simulated network; (**c**) A uniformly sampling scheme in simulated networks; (**d**) A non-uniform sampling scheme in simulated networks; (**e**) The Gini Index of the scheme in Figure 2c; (**f**) The Gini Index of the scheme in Figure 2d.

Gini index is often used for evaluating data inequality according to frequency distributions. There is a demonstration of Gini Index shown in Figure 2a, where *l*_1_ represents the perfect equal cumulative ratio, *l*_2_ represents the actual cumulative ratio, and the area of shaded part *F* represents the Gini Index. If *l*_2_ got closer to *l*_1_, the area of *F*, the Gini Index, will get smaller. In our method, given the sampling nodes number *n* (determined by the sampling rate) of one data collection round, the node sampling scheme can be denoted by a binary vector **q** = (*q*_1_, …, *q_N_*)*^T^*, which is a column of **Q** in Equation (2), where *q_i_* = 1 representing the *i*th node is selected for sensing data and ${\text{∑}}_{i=1}^{N}q_{i}=n$. For each unselected node in the network, we denote the distance of the node to its nearest selected node by *d_j_*. Then we sort the distances and get an ascending sequence *d*_1_ ≤ *d*_2_ ≤ … ≤ *d_N−n_*. From the sequence, we define the evenness measurement for the desired random sampling scheme by Gini Index in the following form:

(16) $$G_{1}(\text{q})=1-\frac{1}{N-n}(2\overset{N-n}{\underset{j=1}{\text{∑}}}d_{j}^{\prime }-1).$$

where $d_{j}^{\prime }\frac{{\text{∑}}_{k=1}^{j}d_{k}}{{\text{∑}}_{i=1}^{N-n}d_{i}}$is the cumulative ratio of distances. By minimization of *G*_1_(**q**), we obtain the optimized q representing the best spatial uniform node selection scheme. As the distances among nodes are unknown for a practical network, we use the spatial correlation weights in Equation (10) to replace the distances *d_j_* and compute the *G*_1_(*q*) in Equation (16). We give a concrete example. As shown in Figure 2. Figure 2b shows a simulated grid network with 21 × 21 node, and Figure 2c,d show two node sampling schemes in the simulated grid network, where the red nodes represent the nodes selected for sensing data. Figure 2e and Figure 2f show their Gini Index of Figure 2c and Figure 2d respectively. Apparently, the area of *F* in Figure 2e is much smaller than the one in Figure 2f as the distribution of the selected nodes in Figure 2c is more uniform than it in Figure 2d. So from the measurement of Gini Index, we could obtain the optimized nodes sampling scheme.

Implementing the data collection round given the node sampling scheme **q**, the energy consumption in the data sensing and delivering will result in a new energy status for the network. We denote the remaining energy of each node by *e_i_*, *i* = 1, …, *N*. They also are sorted as an ascending sequence *e*_1_ ≤ *e*_2_ ≤ … ≤ *e_N_*. Similarly, we define the measurements for the network energy balance by Gini Index in the following form:

(17) $$G_{2}(\text{q})=1-\frac{1}{N}(2\overset{N}{\underset{i=1}{\text{∑}}}e_{i}^{\prime }-1).$$

where $e_{i}^{\prime }\frac{{\text{∑}}_{k=1}^{i}e_{k}}{{\text{∑}}_{j=1}^{N}e_{j}}$.

From Equations (16) and (17), we get a multi-objective 0-1 programming model to search the energy efficient node sampling **q** as the following form:

(18) $$\begin{array}{cccc} \min & Z=\{G_{1}(q),G_{2}(q)\}, & \text{s}.\text{t}. & 1^{T}q=n. \end{array}$$

This is a non-linear optimization problem. Considering the computation restriction in WSNs, we use the intelligent optimization method, Particle Swarm Optimization (PSO) [36], to solve this optimization model. Once obtaining the optimized node sampling scheme **q**, we can implement the current data collection round and so repeat the procedure getting continuous sensing data for low rank recovery at the sink node.

## 6. Experiments and Results

To evaluate the proposed method, we conduct data collection and data recovery experiments on both a simulated network and a real wireless sensor network and compare our method with other relevant methods. In this section, we first give the experiments setup and then report the experimental results.

### 6.1. Experiment Environments and Parameter Setting

#### 6.1.1. The Structure of the Simulated and Real Networks

**Figure 3.**
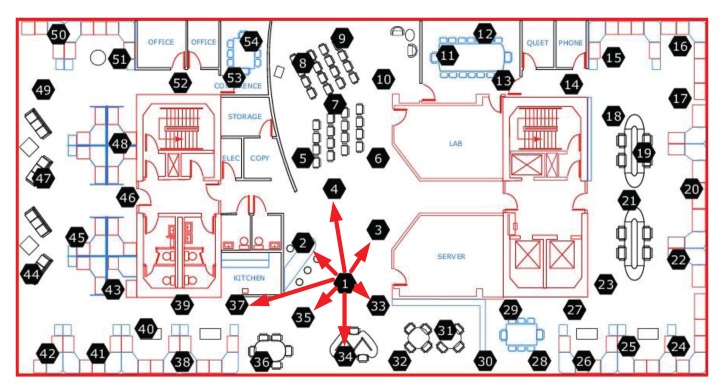
The wireless network of Intel Berkeley Research Lab [37].

The simulated network is a regular 21 × 21 grid network, and the center node is set as the sink node, shown in Figure 2a. In the simulated network, the physical quantities can be accurately sensed and compared with the recovered data, so the proposed method can be evaluated confidently. The real data collection scenario is the wireless network of Intel Berkeley Research Lab [37], which has 54 nodes and the 5th node is the sink node, shown in Figure 3. The sensing data of the real network include several physical quantities, such as temperature, humidity and illumination, *etc.* The data were collected over several months with a sensing time slot of half minute.

#### 6.1.2. The MAC Protocol and Routing of the Simulated and Real Networks

To the regular grid of the simulated network, the experiments are implemented on a scheduled medium access control (MAC). Specifically, IEEE 802.15.4 is adopted as the MAC protocol. The data transmission rate is set 250 kbps. To the nodes irregularly deployed in the real network, the experiments are constructed with 433 MHz mica2dot nodes which adopt the default CSMA based B-MAC as the MAC layer protocol.

To model the sensing data delivering procedure, we adopt a simple routing method to transport a sensing packet from a node to the sink node, in which a node selects the one-hop adjacent node with maximal remaining energy to transmit the sensing packet. In the simulated network, the one-hop adjacent nodes of a node are the directly linked nodes in the grid. In the real networks, we set a maximal distance for a node to transmit data package to others in one-hop. In our experiments, the upper bound is assigned to 10 m. Under this setting, we obtain the connection of the nodes in the real network. For example, the one-hop links from node 1 are shown as the arrowed lines in Figure 3.

#### 6.1.3. The Energy Consumption Model of the Simulated and Real Networks

To evaluate the energy consumption of the network, we adopt the energy consumption model in [38] in our experiments, in which the node transmitting and reception energy consumption *E_TX_* (*d*) and *E_RX_* are defined as follows:

(19) $$E_{T_{x}}(d)=E_{l}+\epsilon_{a}d^{2}.$$

(20) $$E_{R_{x}}=E_{l}.$$

where *d* is the transmitting distance, *E_l_* is the energy consumed of radio electronics, ∈*_a_* is the power amplifier. In our experiments, all nodes are initialized with 2 × 10^4^ *J* of energy and the parameters *E_l_* = 50*nJ*/*bit, ∈_a_* = 10*pJ*/*bit*/*m*^2^. The sensing packet is assumed to be a fixed size of 1000 bits.

Based on the above energy consumption model, given a node sampling rate, the network lifetime is defined as the maximal data collection rounds if the network energy can ensure the sensing data being transferred to the sink node.

#### 6.1.4. Data Recovery Parameters Setup

**Figure 4.**
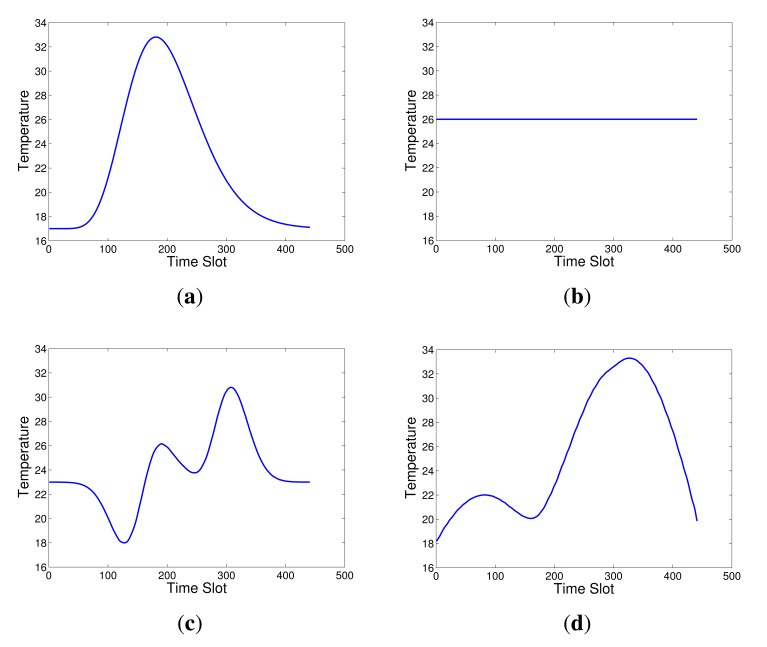
The simulated sensing data of four corner nodes **a, b, c, d** of the simulated network in Figure 2a.

**Figure 5.**
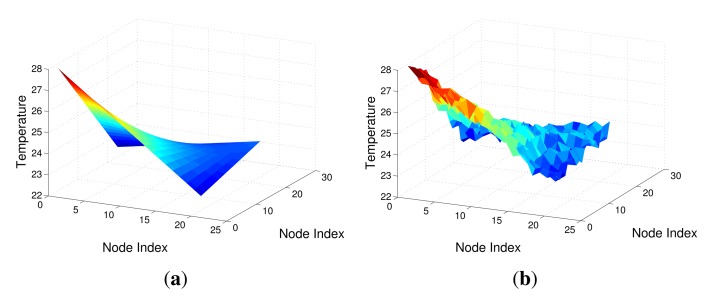
A snap of the sensing data of the simulated network. (**a**) The clean sensing data; (**b**) The sensing data with noise.

In the simulated network, to simulate the change of a physical quantity, such as temperature, we use four curves, shown as Figure 4, to represent the sensing data during a period of time corresponding to the four corner nodes in Figure 2a. The sensing data of other nodes are then generated by the bilinear interpolation method. A snap of the simulated measurements of the 21 × 21 grid is shown in Figure 5a. These clean sensing data are regarded as the ground truth of the physical quantity. However, the real sensing data usually have some noise, so we add Gaussian noise into the clean sensing data to simulate actual measurement data, shown as Figure 5b. To form the measurement matrix **X** for data recovery, here **X** is set as a square matrix with dimension of (21 × 21 − 1) × 440, *i.e., N* = *T* = 440. The parameters in Equations (9), (10) and (15) are empirically set as: λ_1_ = λ_2_ = 0.01, λ_3_ = 10, *α* = 0.1, *ε* = 10^−5^.

**Figure 6.**
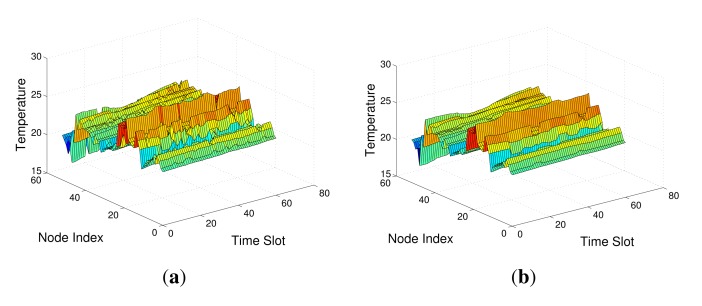
The temperature sensing data from Intel Berkeley Research Lab: (**a**) The actual sensing data; (**b**) The filtered sensing data.

In the real network, the temperature sensing data collected on 53 nodes at March 1st 2004 are used as the measurement data which has dimension of 53 × 2880. As there exist large difference for the dimensions of the row and column, we segment the measurement data into 48 blocks, where each block has same dimension of 53 × 60. One sample of these block is shown in Figure 6a. In the data recovery experiments, each block is assigned as the measurements matrix **X**, *i.e., N* = 53, *T* = 60. The parameters in Equations (9), (10) and (15) are set as: λ_1_ = λ_2_ = 0.01, λ_3_ = 10, *α* = 0.1, *ε* = 10^−5^. The whole recovered data can be obtained by stacking all the recovered blocks.

To evaluate the accuracy of the recovered measurement, the recovery error is defined as the average relative error between the recovered data and the clean sensing data (denoted by **X**^0^) at these unknown element with the following representation:

(21) $$\mathrm{err}=\frac{\sqrt{\underset{\left(i,j)\in \{(i,j)|Q(i,j)=0\right\}}{\text{∑}}\left(\text{X}*(i,j)-{\text{X}}^{0}(i,j)\right)2}}{\sqrt{\underset{\left(i,j)\in \{(i,j)|\text{Q}(i,j)=0\right\}}{\text{∑}}{\left({\text{X}}^{0}(i,j)\right)}^{2}}}.$$

In real networks experiments, we need real clean sensing data to verify the validity of our propose ST-LRMA method. Unfortunately, the actual sampling sensing data are always corrupted and some values are gone missing. The real and accurate temperature data curve should be smooth and continuous, however, the actual temperature sensing data sampled over real networks are always noisy due to one reason or another. Hence, we simply use the Median filter on the temporal direction of the measurements as the clean sensing data to compute the error, *i.e.*, a 1D Median filter with dimension of 5 is used on each row of **X**. The filtered results of the measurements in Figure 6a are shown in Figure 6b. Additionally, for the 48 segmented blocks in the real network, the recovery error is defined as the mean of the recovery errors of the 48 blocks.

To verify the performance of the proposed method on different node sampling rate, defined as the ratio of the number of the valid elements of **M** to the number of the complete elements of the measurement **X**, the data recovery experiments are conducted with the node sampling rate changing from 5% to 95%.

**Figure 7.**
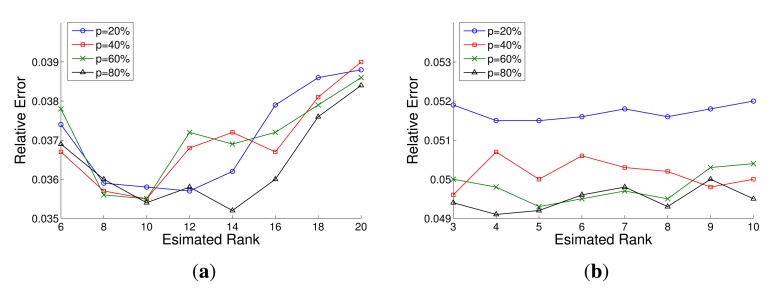
The data recovery results based on different estimated rank and different sampling rate. (**a**) The results in the simulated network; (**b**) The results in the real network.

The rank of the measurements matrix is an important parameter for the LRMA method. However, estimation of the rank from an incomplete matrix is an open problem. To get proper rank estimation, we implement many times data recovery experiments with different rank. In addition, the data recovery results are computed by the above metric. The results of simulative and real networks are shown in Figure 7. According to the results, we choose different rank for the data recovery. In the simulated networks, the appropriate rank is assigned to 10 according to the experimental results shown in Figure 7a. In real network, the appropriate rank is assigned to 5 according to the experimental results shown in Figure 7b.

### 6.2. The Result of the ST-LRMA Based Data Recovery

**Figure 8.**
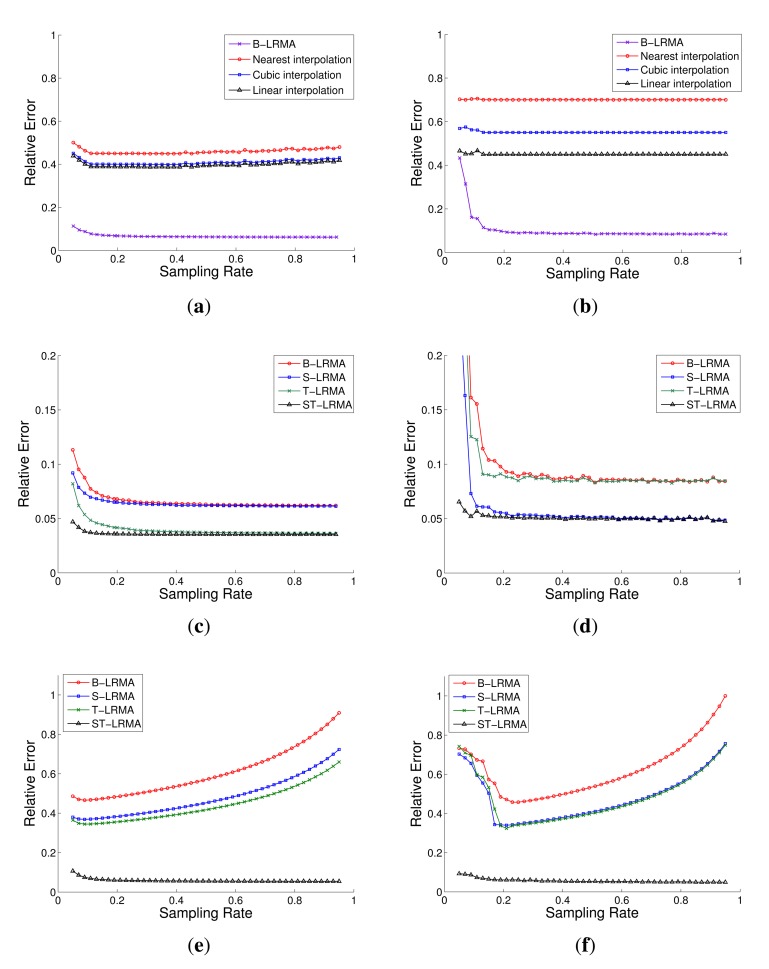
Data recovery results in the simulated and real networks: (**a**) B-LRMA compared with the interpolation methods in the simulated network; (**b**) B-LRMA compared with the interpolation methods in the real network; (**c**) ST-LRMA compared with B-LRMA, S-LRMA and T-LRMA in the simulated network; (**d**) ST-LRMA compared with B-LRMA, S-LRMA and T-LRMA in the real network; (**e**) ST-LRMA compared with B-LRMA, S-LRMA and T-LRMA in the simulated network with the measurement **X** missing rows and columns; (**f**) ST-LRMA compared with B-LRMA, S-LRMA and T-LRMA in the real network with the measurement **X** missing rows and columns.

The proposed ST-LRMA based method are compared with the conventional interpolation methods (linear, cubic and nearest interpolation), the recently proposed LRMA based methods, B-LRMA [14] and T-LRMA [16], and the LRMA based method with only spatial constraint, denoted by S-LRMA. The results, shown in Figure 8a–d, indicate that the LRMA based methods have dramatic improvement in data recovery accuracy compared the conventional interpolation methods. Moreover, the proposed ST-LRMA method outperforms the other LRMA based methods. Especially in the low sampling rate, the proposed method shows a more impressive superiority. This means the proposed method needs only fairly few measurements to obtain high data recovery accuracy. It can be regarded as an energy efficient data collection method and this can be also proved by the following optimized sampling data collection experiments.

According to the low rank completion theory [13], to recover a complete matrix, there should be at least one valid entry at each row and column. However, the data loss often occurs in WSNs. So this condition for the LRMA model may not be guaranteed at some data measurements. To further test the robust of the proposed method, we implement the data recovery experiments with element lost in entire rows and columns of the measurement matrix. Here we randomly remove 10% of rows and columns from the measurement matrices **X** both in the simulated and real networks. Then the above data recovery experiments are repeated using the new inputs. The experimental results, shown in Figure 8e,f, show the proposed ST-LRMA has stable recovery results, while B-LRMA, S-LRMA and T-LRMA generate large error and their curves behave un-convergently. It is considered that the improved stability of the proposed method is due to the newly introduced consistency constraints over measurements.

### 6.3. The Result of the Optimized Sampling for Energy Efficient Data Collection

**Figure 9.**
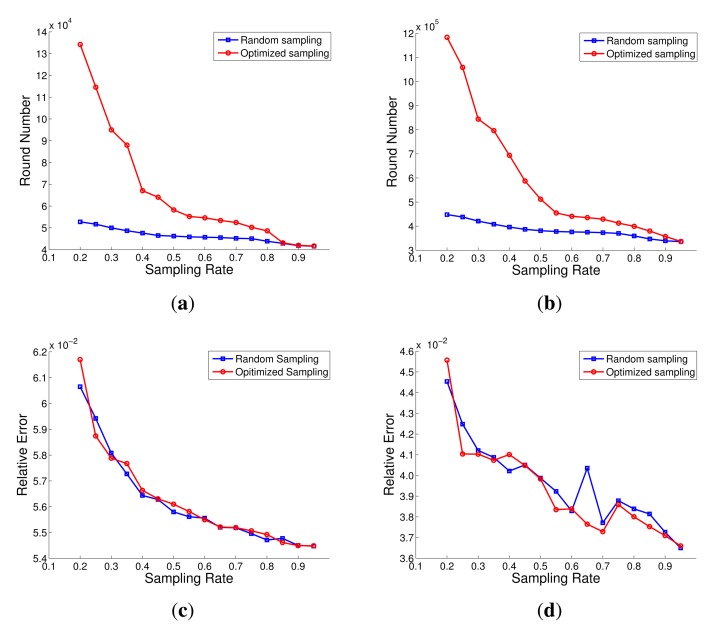
The optimized node sampling method compared with random sampling method. (**a**) The network lifetime of the two sampling methods with different sampling rates in the simulated network; (**b**) The network lifetime of the two sampling methods with different sampling rates in the real network; (**c**) The data recovery error of the two sampling methods with different sampling rates in the simulated network; (**d**) The data recovery error of the two sampling methods with different sampling rates in the real network.

To evaluate performance of the optimized node sampling method, we implement data collection in the whole network lifetime with different node sampling rate. The results are compared with the random sampling method, as shown in Figure 9a for the simulated network and Figure 9b for the real network, respectively. It is shown that the optimized node sampling method has better performance than the random sampling method for both the simulated and real networks. Especially in the lower sampling rate, the optimized node sampling method could obtain better network energy distribution and prolong the network lifetime. For example, when the sampling rate is lower than 30%, the network lifetime of the optimized method will be prolonged two times than the network lifetime of the random method.

The data recovery errors are also computed for the two node sampling methods and the results are reported in Figure 9c,d. In the simulated network or the real network, the optimized node sampling method almost has the same performance to the ideal random sampling method which is considered the best way to get good recovery results. From these, we can conclude that the optimized method can not only extend the network lifetime but also keep the accuracy of the data recovery. This is due to the balanced model concerning the node uniform sampling and the energy distribution of network in Section 5.

## 7. Conclusion

In this paper, we propose an efficient data collection method based on LRMA for WSNs. The contribution of the work lies in two folds. On one hand, a proposed novel ST-LRMA based data collection method introduces the spatial and temporal correlation of the sensing data into the conventional LRMA model. The experiment results indicate that the proposed method has better data recovery accuracy compared with the conventional interpolation methods and the state-of-the-art LRMA based methods. In addition, the proposed method avoids the optimization problem involving empty columns or rows, and could fill the empty columns or rows stably. On the other hand, an optimized nodes sampling method is proposed and integrated with the ST-LRMA based data collection method. In the proposed nodes sampling model, Gini index is adopted to measure the uniformity of the selected nodes and the distribution of the network energy. The data collection experiments were conducted on the simulated and real wireless networks. The results show the proposed method has good performance on network energy efficiency without losing much data recovery accuracy.

Our experiments are implemented without considering some network properties, such as the complex routing, the network time duty circle, and the sensing data attributes. The future work is to integrate these network properties into our ST-LRMA based data collection method and apply it on real large scale wireless sensor networks. Another possible future work is to consider the multi-modality sensing data together and to formulate the sensing data as tensors to be recovered by low rank alike constraints.
